# Application of Machine Learning Methods for an Analysis of E-Nose Multidimensional Signals in Wastewater Treatment

**DOI:** 10.3390/s23010487

**Published:** 2023-01-02

**Authors:** Magdalena Piłat-Rożek, Ewa Łazuka, Dariusz Majerek, Bartosz Szeląg, Sylwia Duda-Saternus, Grzegorz Łagód

**Affiliations:** 1 Faculty of Technology Fundamentals, Lublin University of Technology, 20-618 Lublin, Poland; 2 Faculty of Environmental, Geomatic and Energy Engineering, Kielce University of Technology, 25-314 Kielce, Poland; 3 Institute of Rural Health in Lublin, 20-090 Lublin, Poland; 4 Faculty of Environmental Engineering, Lublin University of Technology, 20-618 Lublin, Poland

**Keywords:** machine learning, multidimensional signals analysis, t-SNE method, k-median method, random forest, electronic nose, gas sensors array, wastewater treatment, wastewater quality

## Abstract

The work represents a successful attempt to combine a gas sensors array with instrumentation (hardware), and machine learning methods as the basis for creating numerical codes (software), together constituting an electronic nose, to correct the classification of the various stages of the wastewater treatment process. To evaluate the multidimensional measurement derived from the gas sensors array, dimensionality reduction was performed using the t-SNE method, which (unlike the commonly used PCA method) preserves the local structure of the data by minimizing the Kullback-Leibler divergence between the two distributions with respect to the location of points on the map. The k-median method was used to evaluate the discretization potential of the collected multidimensional data. It showed that observations from different stages of the wastewater treatment process have varying chemical fingerprints. In the final stage of data analysis, a supervised machine learning method, in the form of a random forest, was used to classify observations based on the measurements from the sensors array. The quality of the resulting model was assessed based on several measures commonly used in classification tasks. All the measures used confirmed that the classification model perfectly assigned classes to the observations from the test set, which also confirmed the absence of model overfitting.

## 1. Introduction

Wastewater treatment plants (WWTPs) are technological objects that reduce the pollution load in wastewater before its discharge to a receiver—mainly surface water [1,2]. In turn, surface water quite often serves as a resource for treatment and preparing potable water which should be monitored [3,4,5]. At present, a major part of the operating of WWTPs operating in developed countries involves the intense implementation of highly efficient methods of mechanical, biological, and chemical treatment of wastewater, mainly with the application of the activated sludge method [1,6,7]. The afore-mentioned method employs the systems with the integrated removal of carbon, nitrogen and phosphorus compounds, in which the treatment process is carried out under aerobic–anaerobic conditions [8,9,10,11]. Municipal wastewater mainly comprises the spent water discharged from houses, public institutions, industrial wastewater, as well as precipitation, seepage, and thaw water. The main groups of pollution found in the considered medium include degradable organic substances, other organic compounds, biogenic elements (i.e., nitrogen and phosphorus), microorganisms, refractive and toxic substances, heavy metals, and other inorganic compounds [12,13]. However, not all pollutants found in water are determined during the wastewater examination, and used for WWTP control, because there are too many of them and the classification of each would be impossible or very expensive. In practice, groups of the most indicative pollution indices helpful for the assessment of a negative impact on the environment are determined. The organic compounds found in the wastewater are determined using the amount of spent oxygen (O_2_) as COD (chemical oxygen demand) or BOD (biochemical oxygen demand), or as the amount of generated carbon dioxide (CO_2_), as TOC (total organic carbon). TSS (Total Suspended Solids) is also an important general pollution indicator [13,14,15].

An electronic nose, which mimics the olfactory sense of mammals [16,17] consists of two basic components, namely a multi-sensor array with instrumentation that yields signals from measurements, and a system for analyzing multidimensional signals obtained from that array [13,18,19,20]. In the case of the electronic nose, it is an array of gas sensors. Each gas sensor is partially sensitive to different groups of chemical compounds. Each gas mixture forms a unique signal profile that may be compared to fingerprints in dactyloscopy, since the formation of an identical combination in two distinct gas samples is extremely unlikely. Therefore, signal combinations are commonly referred to as “gas fingerprints” [19,21,22],. This approach is generally similar with other kinds of signal analyses, e.g., “fingerprint IR” or slope [23,24]. Fingerprints can also be analyzed in reference to their parameters, e.g., as “slope fingerprints” or “similarity fingerprints”. They are connected with methodology known as Fluctuation Enhanced Sensing (FES) that can enhance the gas detection and classification even using small number of sensors [25,26]. Because the multi-sensor array yields a set of signals that are virtually unique, it is possible to accurately distinguish the gas mixture samples under investigation. The sensors used in this case should be sensitive to different groups of contaminants.

The sensors used in electronic noses comprise metal oxide semiconductor sensors (MOS), conductive polymers (CP), quartz crystal microbalance (QCM), or surface acoustic wave (SAW). MOS sensors are commonly used in the arrays employed in environmental engineering; they usually involve tin dioxide (SnO_2_) with such additives as platinum, gold and silver (added to enhance the selectivity of the gas-sensitive layer) [20,27]. Chemisorption takes place on the surface of the sinter. The electrons of the gas and the semiconductor form a bond, changing electrical conductivity, and enabling measurements to be conducted [28]. Depending on the type of sensors used, they can, to a certain extent, distinguish between the individual components or groups of components present in the mixture under consideration, making it possible to assign different parameters to the readings [13]. Since electronic noses were designed to analyze and classify gaseous mixtures, their first applications in analyzing the performance of water and wastewater management facilities were primarily related to classifying the odor nuisance of wastewater and wastewater treatment facilities [21,29,30,31,32].

Electronic noses can give the information on the features related to water or wastewater quality by analyzing the headspace. This is made possible by Henry’s law. At a constant temperature, the concentration of certain compounds in the C_G_ gas phase, which is in equilibrium with the liquid, is directly proportional to the concentration in the liquid phase. This relationship is described by the H = C_G_/C_V_ equation, enabling the concentration of organic and mineral compounds in wastewater to be evaluated via headspace analysis [33]. Hence, e-noses can be used to analyze, evaluate, and classify the level of contaminants present in water and wastewater [20,34,35,36,37,38].

The above-mentioned works confirm that gas-based multisensor arrays can be used to monitor the processes taking place during wastewater treatment under laboratory conditions and at technical-scale facilities. Some works have also focused on identifying the presence of crude oil derivatives, pesticides, and other chemical compounds in the influent of WWTPs, which can be detrimental to activated sludge and disrupt biological treatment processes. Some papers have shown the possibility of classifying or evaluating the quality of treated or laboratory-prepared wastewater. Nevertheless, the analysis and evaluation of all primary stages of operation of a full-scale WWTP, i.e., mechanically treated wastewater, subjected to high-efficiency treatment in activated sludge bioreactors at the treatment plants where odor nuisance is practically non-existent, is poorly represented in the literature [13].

Moreover, the literature lacks the studies that present consecutive steps in data analysis, including their initial interpretation and visualization related to determining the potential for appropriate classification. The next step is to demonstrate clustering in a multidimensional space using unsupervised learning methods. The final step is the application of a supervised method to classify as accurately as possible the multidimensional signals from a matrix of gas sensors, which would uniquely identify sampling points located in the treatment plant. Thus, the paper is a presentation of the procedure for dealing with multidimensional data, together with an indication of possible machine learning methods and possibly extensive references to the literature on the subject. As part of the work, the authors presented a three-step method of data analysis:

1. The t-SNE method for visualizing and reducing the dimensionality of the data;
2. The k-median method to seek general relationships and the relationships between groups of data in multidimensional space;
3. The random forest model for the final classification of observations as well as the identification of data sets.

## 2. Review of Advances in Machine Learning Methods for Analysis of Multidimensional Data

In order to adequately interpret the multidimensional data sets derived from successive readings taken with a gas sensor array, advanced statistical methods are needed. When dealing with this type of issue, the methods to reduce the number of dimensions and visualize the similarities present between the analyzed samples, e.g., principal component analysis (PCA), are usually applied at the beginning. This type of statistical method involves grouping primary data using a new low-dimensional space generated by linear combinations of primal variables. When the dimensionality of the original space is reduced, the data can then be represented in graphs [39,40]. Due to the transformations, the original information is partially lost in favor of a simpler data structure [41,42]. The use of unsupervised machine learning methods can indicate classification and similarities between data by operating on the observations made in multidimensional spaces [43,44]. Another possible way to analyze the multidimensional data obtained from gas sensor arrays is to use supervised machine learning methods.

Supervised learning techniques can confirm that the aforementioned hidden structure (homogeneous clusters of data) can be applied for classification purposes. Any tested system may be described using a set of classifications with both input and output parameters. Environmental states, preconditions, and other rather uncommon parameters can also constitute input parameters [45,46]. Each classification may involve any number of disjointed classes, which describe the occurrence of a given parameter. Classes are usually selected by conforming to the principle of equivalence partitioning for abstract test cases and boundary-value analysis for specific test cases [47]. Classifications can be grouped into compositions for semantic purposes.

With this information in mind, the purpose of this paper is to demonstrate the possibility of classifying and hence assessing the quality of treated wastewater in a full-scale municipal WWTP by finding a hidden structure in the multidimensional space generated from gas sensor readings. This structure was found using unsupervised machine learning methods. The state of current knowledge of electronic nose applications does not allow the use of deterministic models for object classification. The relationships between readings from individual sensors and membership in the right class are characterized by high complexity. At the same time, in the family of classical machine learning models, such as multinomial logistic regression, linear discriminant analysis or single decision trees, the obtained solutions leave much to be desired [48,49,50,51]. Therefore, advanced machine learning models such as SVM, RF, or ANN are necessary to properly classify objects based on statistical models [52,53,54,55,56]. In addition, since the readings from the electronic nose do not have reference values, an analysis of the input signals can only be completed by comparing measurements between groups. For this purpose, cluster analysis is used to determine homogeneous groups of observations. In turn, t-SNE analysis allows dimensionality reduction and then organoleptically assesses the quality of the resulting groups. Supervised learning techniques, i.e., Random Forest, confirmed that the aforementioned hidden structure (homogeneous clusters of data) can be applied to classify wastewater at different stages of the treatment process. To the best of authors’ knowledge based on the literature analysis, this type of multistage analysis of multidimensional data from gas sensor arrays evaluating the wastewater collected in successive stages of wastewater treatment plants, as well as for any other environmental engineering object, has not been described before.

Since the original dataset consisted of readings from 17 sensors, it was necessary to use a method that would allow interpretation of the results based on a scatter plot. Such methods include, for example, PCA (*Principal Component Analysis*) first presented in [57], various types of MDS (*Multidimensional Scaling*) and t-SNE (*t-Distributed Stochastic Neighbor Embedding*), which was presented in [58]. These methods are also widely used in the applications related to environmental engineering and, in particular, wastewater treatment plants [13,59]. PCA is a also widely used method in visualizing readings from e-noses and other electronic sensing devices; it has been applied in [13,60,61]. In this work, the t-SNE method was adopted because of its properties, which usually allow visualizing the data in a two- or three-dimensional space, avoiding the concentration of all points in the center of the graph.

The t-SNE algorithm is based on the SNE (Stochastic Neighbor Embedding) method presented in [62]. In the SNE method, for each element of the set *i*, the asymmetric probability of selecting element *j* as a neighbor is calculated:

(1) $$p_{i|j}=\frac{e^{-d_{ij}^{2}}}{\sum_{k\neq i\;}e^{-d_{ik}^{2}}}.\;$$

Function $d_{ij}$ is the distance between $x_{i},\;x_{j}$, which are *n*-dimensional elements. Thus, it is usually calculated as a scaled distance in Euclidean metric:

(2) $$d_{ij}=\frac{\left|\left|x_{i}-x_{j}\right|\right|}{\sqrt{2}\sigma_{i}},$$

where $\sigma_{i}$ denotes the entropy of the probability distribution of neighbors. In the case of low-dimensional spaces, the variance can be predetermined, equal to $\frac{1}{2}$. The method also calculates the induced probabilities $q_{i|j}$, which use images of points $x_{i},\;x_{j}$, that is, the values of the explained variables $y_{i},\;y_{j}$:

(3) $$q_{i|j}=\frac{e^{-{\left|\left|y_{i}-y_{j}\right|\right|}^{2}}}{\sum_{k\neq i\;}e^{-{\left|\left|y_{i}-y_{k}\right|\right|}^{2}}}.$$

The goal of SNE is to best fit the distributions $p_{i|j}$ and $q_{i|j}$, which is to minimize the Kullback-Leibler divergence [63] of the form:

(4) $$C=\underset{i}{\sum }D_{KL}(P||Q)=\underset{i}{\sum }\underset{j}{\sum }p_{i|j}\cdot \log\frac{p_{i|j}}{q_{i|j}}.$$

Hence, the goal is to differentiate this function by the variable $y_{i}$, which results in the formula:

(5) $$\frac{\partial C}{\partial y_{i}}=2\underset{j}{\sum }\left(y_{i}-y_{j}\right)\left(p_{i|j}-q_{i|j}+p_{j|i}-q_{i|j}\right).\;$$

The cost function in SNE is quite difficult to minimize due to its form. For this reason, the t-SNE algorithm was developed, which is its symmetric modification [58]. The probability of choosing element *i* as a neighbor of element *j* is the same as choosing *j* as a neighbor for element *I*; hence for each element i,j the conditions $p_{ij}=p_{ji},\;q_{ij}=q_{ji}\;$and $p_{ii}=q_{ii}=0$ are satisfied.

Thus, these probabilities are defined as:

(6) $$p_{ij}=\frac{e^{-\frac{{\left|\left|x_{i}-x_{j}\right|\right|}^{2}}{2\sigma^{2}}}}{\sum_{k\neq l\;}e^{-\frac{{\left|\left|x_{k}-x_{l}\right|\right|}^{2}}{2\sigma^{2}}}},$$

In turn, for the calculation of induced probabilities in this case, Student’s t distribution with 1 degree of freedom is used. Then $q_{ij}$ are expressed by the formula:

(7) $$q_{ij}=\frac{1}{\sum_{k\neq l\;}\frac{1+{\left|\left|y_{i}-y_{j}\right|\right|}^{2}}{1+{\left|\left|y_{k}-y_{l}\right|\right|}^{2}}}.\;$$

Then, the cost function can be written as:

(8) $$\tilde{C}=\underset{i}{\sum }D_{KL}(P||Q)=\underset{i}{\sum }\underset{j}{\sum }p_{ij}\cdot \log\frac{p_{ij}}{q_{ij}},\;$$

whereas its derivative is expressed as:

(9) $$\frac{\partial \tilde{C}}{\partial y_{i}}=4\underset{j}{\sum }\frac{\left(p_{ij}-q_{ij}\right)\left(y_{i}-y_{j}\right)}{1+{\left|\left|y_{i}-y_{j}\right|\right|}^{2}}.\;$$

The form of the cost function presented above means that the points that have significantly different values of variables from each other will lie far apart on the plane. The t-SNE method is widely used in medical applications, such as visualization of RNA sequencing from single cells [64], graphical representation of the human genome [65], or projection of metagenomic contigs from the mouse gut microbiome [66].

Cluster analysis is another method that can show the ability to create homogeneous groups in data. The foundations of this method were presented in [67]. It constitutes a set of statistical methods that aim at creating disparate groups from a set of data. The main division in cluster analysis is between hierarchical and non-hierarchical methods, but the methods based on density function, or fuzzy clustering can also be distinguished. Non-hierarchical methods are based on a matrix of distances between points in space, calculated for a selected metric [43]. Some of the most popular cluster analysis algorithms are the k-means and k-median algorithms. Both of these algorithms need a fixed parameter *k* to operate, which is the number of groups formed from the input dataset. In the k-means algorithm, the arithmetic mean of the observations belonging to a given group is used as the similarity parameter between the observations, while in k-median it is the median of the observations [68]. Both of these algorithms operate in the following way: at the beginning of the operation, *k* observations are drawn, which are the first means of the clusters, then using the distance matrix in the selected metric, objects are assigned to the corresponding clusters and new cluster centers are determined. This procedure, in addition to the first step, is repeated until there are no shifts between clusters, or a stop condition is reached, which can be a predetermined number of iterations. Cluster analysis has been used to classify the factors affecting water quality in publications [69,70], while methods of k-median and k-means were used in the work [71] to identify the size of airborne pollutant particles.

Once the clustering ability of data is confirmed, the next step is to create a classification model that will assign observations to the above-mentioned groups. Supervised machine learning models are used for this purpose. Such models can be classification trees [72], random forests [73], or neural networks [27]. Classification trees were used in [13] to identify wastewater treatment stages and in [74,75] to detect bulking sludge in activated sludge. In turn, artificial neural networks were used in [75,76,77,78] related to the classification and prediction of the occurrence of substances in activated sludge. In this paper, a classification model is built using the random forests algorithm described in [79,80]. This method involves using a large number of decision trees to solve a machine learning task. Random forests are a combination of tree predictors, such that each tree depends on the value of a random vector $\Theta_{k}$, which is drawn independently of the values of previous vectors $\Theta_{1},\ldots ,\Theta_{k-1}$, according to an identical distribution for all trees in the forest. Each tree is built using the learning set and the corresponding vector $\Theta_{k}$. In this way, a classifier $h\left(x,\;\Theta_{k}\right)$, is obtained, where x is a vector of input data. Each tree created in this way casts a unit “vote” for the most likely class given the input data x.

The first approach to the construction of random forests was presented in [81], where a random selection is made to build each tree without returning observations from the test set. Another approach presented in [82] was to randomly select a split from among the *K* best split rules at a given node. In [83], generating new learning sets by randomizing the resulting values from the original learning set was proposed. However, currently the most widely used approach is the one presented in [84], in which m variables are randomly selected for each tree built based on a learning set with n explanatory variables. For classification, it is most often assumed that $m=\sqrt{n}$ while in the case of a regression task, $m=\frac{n}{3}$. Building the model in this way reduces the risk of over-correlation of predictions obtained from individual trees, because by the randomness of the selection of variables, those that most strongly affect the prediction from the model will not be involved in the construction of each tree. The generalization error of random forests converges almost surely to its limit when the number of trees in the forest tends to infinity, regardless of their construction. Random forests were used to identify bulking sludge in the work [75].

Traditional approaches typically use a confusion matrix and accuracy to evaluate model performance. Accuracy is one of the most straightforward metrics used in machine learning. It determines how accurate a model is, but it does not indicate which class is classified best and worst. For this reason, other metrics, most commonly found in binary classification, were introduced to assess model fit.

Cohen’s Kappa is defined as

(10) $$\kappa =\frac{p_{0}-p_{e}}{1-p_{e}},\;$$

where $p_{0}$ is observed agreement, and $p_{e}$ is the expected agreement. It tells how much better a given classifier is performing over the performance of a classifier that simply guesses at random according to the frequency of each class. Other two very important measures are

(11) $$Precision=\frac{TP}{TP+FP},\;\;\;\;\;\;\;\;\;\;\;\;\;\;\;\;Recall=\frac{TP}{TP+FN}$$

Precision (also called positive predictive value) is the fraction of relevant instances among the retrieved instances, while recall (also known as sensitivity) is the fraction of relevant instances that were retrieved. To evaluate model performance comprehensively, both precision and recall should be examined. The F1 score serves as a helpful metric that considers both of them

(12) $$F1=2\frac{Precision\cdot Recall}{Precision+Recall}\;.\;$$

The multiclass implementations use micro, macro, and macro-weighted averaging where applicable, and some metrics have their own specialized multiclass implementations [85,86]. In this paper, macro-weighted averaging was applied, because of a slight imbalance was observed in test set. Macro-weighted averaging involves calculating a weighted average of the desired measure in a one-to-all approach. In addition, ROC curves were plotted for each class, and the AUC was determined by averaging all areas according to the Hand-Till method [86].

## 3. Materials and Methods

The wastewater under consideration was collected from the “Hajdów” Municipal Wastewater Treatment Plant in Lublin (Southeast Poland), with an average daily wastewater volume of 60,000 m^3^ d^–1^. This mechanical–biological treatment plant operates in continuous flow mode, and a modified Bardenpho [13,87] was used in the bioreactor chambers. The samples were collected directly from the technological equipment in the biological and mechanical parts at five points: the primary settling tank, mixing chamber, bioreactor inlet and outlet as well as secondary settling tanks (treated wastewater). The wastewater samples were collected in bottles, filled and promptly taken to the laboratory for analysis. The time from sample collection to analysis was about 30–45 min. During transport, the bottles were stored in a travel refrigerator.

A self-constructed gas sensor array consisting of 17 Figaro MOS sensors, described in detail in previous works by a team from Lublin University of Technology [13,33], was used for the measurements. The sensors used were characterized by their low power consumption and small size [88]. The measurements were conducted in a 3–5 arrangement for each port, including 3 min of sensor flushing with synthetic air and 5 min of analyzing the mixture.

A homogeneous composition was obtained after intensive stirring. The initial samples of the medium under consideration (100 mL) were poured in equal amounts into three identical glass conical flasks and then analyzed by means of a multi-sensor gas matrix. The procedure was carried out in triplicate. Between measurements, the flasks were rinsed several times with distilled water.

In addition, to reference the array readings and to determine the level of contamination of subsequent samples, total organic carbon–which is one of the basic and most commonly measured parameters–was determined via catalytic oxidation, by means of a TOC 5050A total organic carbon analyzer (Shimadzu, Kyoto, Japan). In addition, total suspended solids (TSS) were determined using a HACH DR 3900 spectrophotometer from HACH-Lange (Hach Lange GmbH, Düsseldorf, Germany) via photometric method 8006 (program 630), in accordance with the protocol recommended by the manufacturing company. The device recorded sensor readings at 5-s intervals, so the resulting data sets were prepared accordingly to average the presented results, reduce the number of analyzed points and improve the readability of the graphs. The initial dataset used for statistical analysis had the total size of 185.

**Figure 1:**
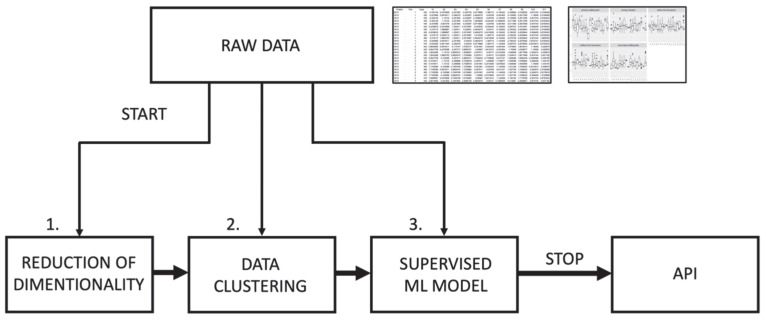
Research diagram.

Figure 1 shows the diagram of research conducted in this paper, which is the multi-step procedure of analyzing raw, multidimensional data for preparing electronic nose software.

All statistical analyses, including the graphs found in this paper, were performed in the language for statistical computing R version 4.2.1 [89] in the programming environment RStudio version 2022.7.0.548 [90]. The functions found in the following libraries of this software were used for the present calculations.

The caret package was created by Max Kuhn et al. and it was released on CRAN (Comprehensive R Archive Network) in 2007, its documentation can be found in [91]. It is used for creating various machine-learning models used for prediction [92]. This package contains the trainControl function, which was used for controlling how the random forest model was trained. The expand.grid function was applied for restricting values of the tuned parameters. The train function allowed training the model on the learning set.

The package named cluster was first published in 1999 by Maechler et al. [93] as a tool for applying cluster analysis methods. In this paper, the pam function was used, the function allows to apply k-medoids method for clustering the original dataset into k disjoint sets. The number k is chosen by the user.

The Rtsne package was first released in 2014 by Krijthe and van der Maaten for implementing the code for T-distributed Stochastic Neighbor Embedding in R programming language [94]. The function Rtsne was used for reducing the number of dimensions in the dataset.

The package tidyverse was created by Wickham and RStudio Team in 2016 [95]. This package loads a number of different packages that may be applied for organizing and visualizing data. The ggplot2 package, which belongs to the tidyverse collection, is one of the most widely used packages [96]. It was used for creating all figures in this paper.

## 4. Results and Discussion

**Figure 2:**
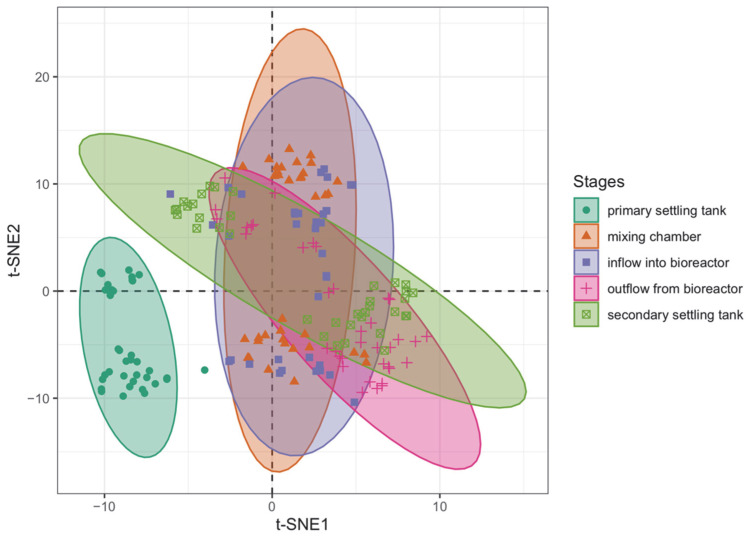
Two-dimensional t-SNE mapping of dimensionally reduced data. Different stages in wastewater treatment process are denoted by distinct colors and ellipses.

Figure 2 shows the results of scaling the 17-dimensional space of explanatory variables containing sensor readings into a two-dimensional space using the t-SNE method. The different-colored ellipses shown in the graph delineate the 95% confidence area for each group established by the different stages of wastewater treatment. In this graph, it is clear that the sample from the primary settling tank stands out significantly from the other samples. The confidence area for these elements did not overlap with the areas containing the other wastewater treatment stages. The fact that the group containing the observations from the secondary settling tank did not overlap with the observations from the primary settling tank enabled us to conclude with high probability that it will be possible to create a model that classifies the observations that are homogeneous with each other into heterogeneous groups.

**Figure 3:**
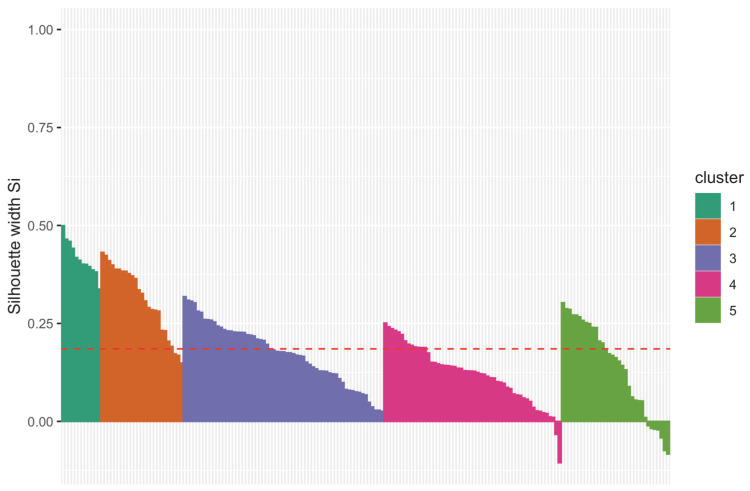
Silhouette plot of clusters obtained from the k-medoid algorithm.

Since it is clear from the previous figure that it was possible to extract some clusters, the k-median clustering algorithm was used to find homogeneous groups. Because the explained variable was divided into five stages of cleaning, the number of clusters into which the data should be split was known beforehand. That is why the parameter k in the algorithm was predetermined k=5. Table 1 presents the basic statistics for each of the clusters formed in the k-median algorithm. In turn, Figure 3 shows silhouette plots of each cluster in the k-median algorithm for k=5. It can be seen that each of the clusters formed had a silhouette score above the average level for all the data. On the basis of this information and external knowledge, it can be concluded that the chosen number of clusters was correct.

**Figure 4:**
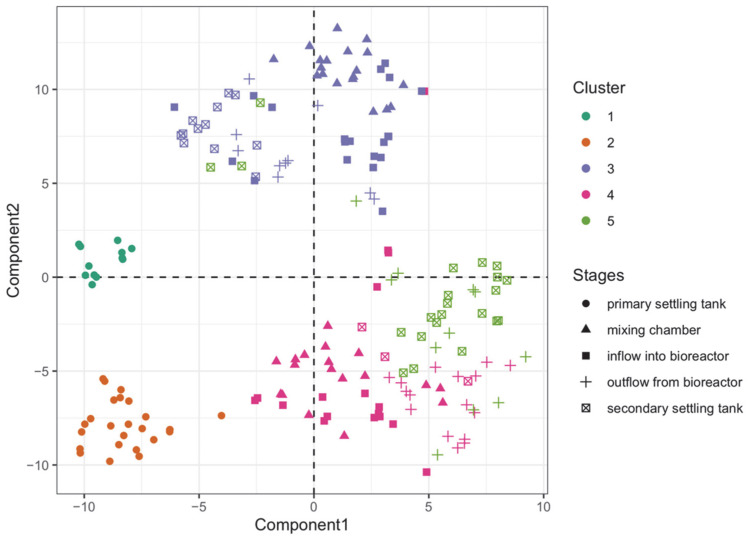
The result of the k-median clustering algorithm. The different shades indicate the clusters into which the observations were classified, while shapes indicate the corresponding stages in the wastewater treatment. The components listed on the graph axes are those created using the t-SNE method.

Figure 4 shows the result of the cluster analysis algorithm using the k-median method in the two-dimensional space built using the t-SNE method. It can be seen that the dark green and orange colors marked the observations from the pre-settler. It can also be seen that the in majority, the light green color marked the observations from the secondary settling tank. Such a result confirms the clustering ability of the data, already noted in Figure 2. The correctness of the classification of this analysis can also be observed in Table 2, which shows that, as a result of the cluster analysis, stage one in the activated sludge treatment was divided into two clusters, which were disconnected from the other observations forming three consecutive groups. In the other three clusters formed, there were incorrectly classified observations. However, it can additionally be concluded that the algorithm noticed the greatest similarities between the observations coming from the secondary settling tank and the bioreactor outflow. This is because the observations from only these samples, which in majority were taken from the secondary settling tank, were classified into cluster 5. Thus, using the k-median cluster analysis method, it can be concluded that it is possible to classify the relevant stages of wastewater treatment using the readings from the 17 matrix sensors.

**Figure 5:**
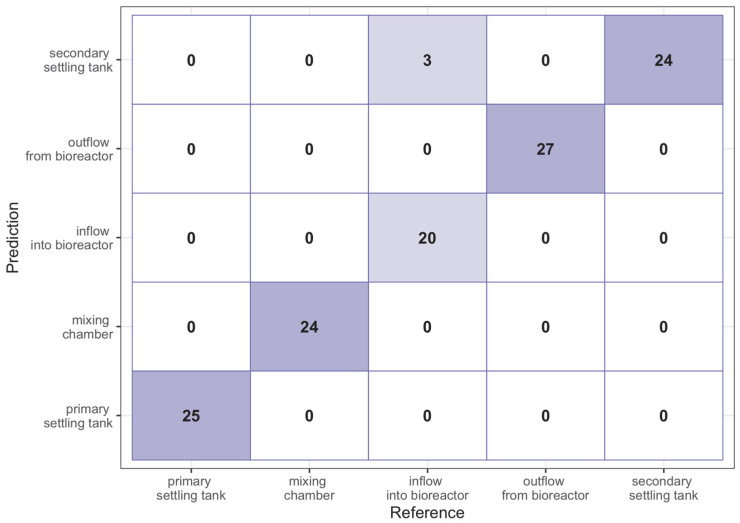
Contingency matrix for random forest model on the learning sample.

To confirm the ability to classify observations into appropriate heterogeneous groups, a supervised machine learning model was used, namely random forest. The original dataset consisted of 185 observations. To create the model, the data were divided into a learning set, which made up $\frac{2}{3}$ of the dataset, and a test set, which formed the remaining observations. The data for the learning set were selected randomly. The parameter mtry denoting the number of variables randomized to each tree was tuned based on a 5-fold cross-check. Due to the moderate size of the learning set, the 5-fold cross validation method was used to assess the quality of the fit. Too small number of observations per fold could prevent proper estimation of hyperparameters. Due to the fact that the parameter mtry =$\;\sqrt{n}$, is usually chosen, this parameter was checked for values from 3 to 5 in order to avoid overtraining the model. This phenomenon can occur if there are too many variables in each of the trees being built. The parameter denoting the number of trees in the random forest was not tuned and this value was left as the default and equal to 500, due to the fact that too many trees will not overtrain the model [97]. As a result of tuning the model, due to the classification accuracy parameter (Accuracy), the value of the parameter mtry=5 was obtained, for which the model on the learning set obtained a high percentage of correct classifications, because it was 97.5%. The random forest model was trained on the learning set for approximately 2.2 s. Figure 5 shows the matrix of correct classifications for this set; the lighter purple color indicates the cases in which the classification was not 100% correct. This shows that the model was wrong for only three observations in the learning set, while these errors were only for the observations from the sample coming from the inflow to the bioreactor. The random forest model classified them as the observations originating from the secondary settling tank.

**Figure 6:**
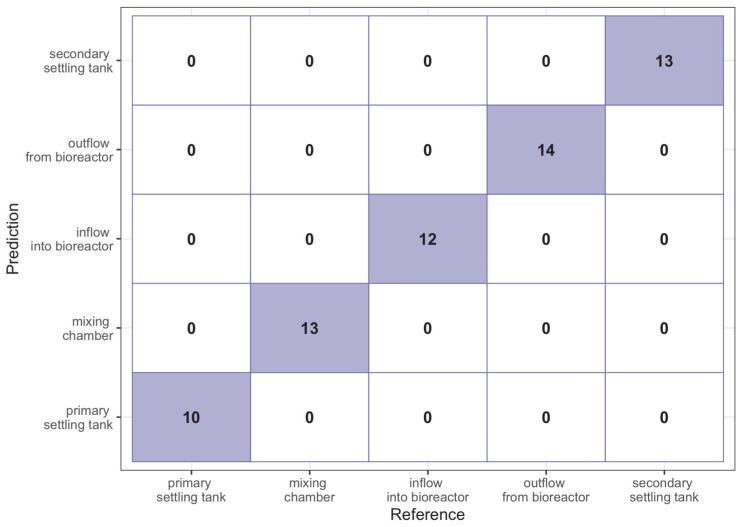
Contingency matrix for random forest model on the test sample.

In turn, Figure 6 presents the contingency matrix for the prediction from the same model to the test sample. As it can be seen, the model made no error on any classification, making the Accuracy parameter equal to 1 for this sample.

**Figure 7:**
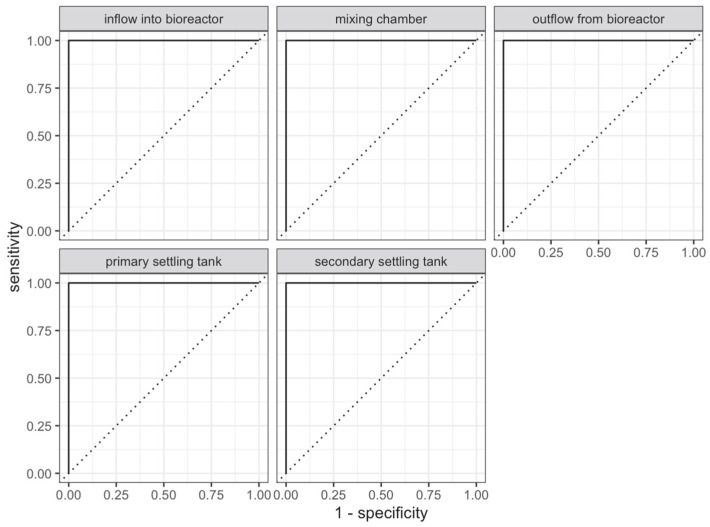
ROC curve of each class on test set.

Additional results on the ROC curves calculated for each class in the test set can be observed in Figure 7 and Table 3. Since the classification on the test set was 100% correct, the results were ideal.

The fact that the random forest model achieved 100% correct classifications on the test set does not mean that the model is free of limitations. The algorithm needs to be recalibrated for each set of input data, which is a significant difficulty in the application of this method.

**Figure 8:**
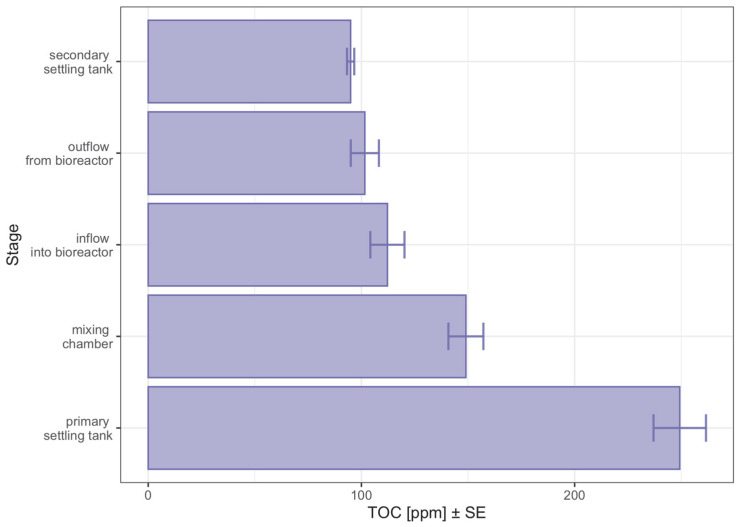
Graph of total organic carbon (TOC) values in the samples tested broken down by the wastewater treatment stages.

In line with Figure 8, the amount of total organic carbon varies depending on the stage of wastewater treatment. The amount of total carbon decreased with the successive stages of treatment and was significantly higher in the primary settling tank. The amount of total organic carbon in the samples from the other stages was similar to each other. A similar observation can be seen in Figure 2, where the observations from the pre-settler were in a separate part of the t-SNE space, not overlapping with the observations from the other treatment stages. This fact can also be seen in Figure 4 and Table 2, where the observations from the pre-settler were classified into the groups that were disjointed from the groups to which the others were assigned. The random forest model, the results of which are shown in Figure 3 and Figure 4 also shows that the observations that represented the samples with untreated water were better classified, relative to the observations which represented the water that had already undergone some treatment. The learning set erroneously classified the observations from the bioreactor influent as a component from the secondary settling tank.

**Figure 9:**
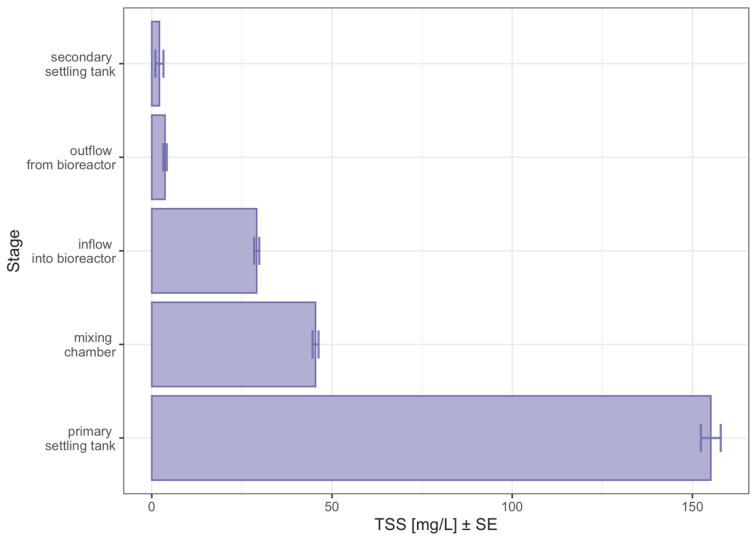
Graph of the level of total suspended solids (TSS) in the samples tested broken down by the wastewater treatment stages.

The overlap of samples from the last four stages of treatment seen in Figure 2 can also be explained by the observation obtained in Figure 9, where the variation of suspended solids content in the treated water is shown. In this graph, it can be seen that the TSS content of the samples was more than three times higher in the pre-settler, relative to the samples from the mixing chamber. This again demonstrates the high discriminatory power of the observations from this stage of wastewater treatment.

## 5. Summary and Conclusions

Performing a rescaling of the 17-dimensional space of variables to a 2-dimensional space using the t-SNE method allows the data on a plane to be visualized and the differences between the elements from various stages of treatment to be noticed.

The k-median cluster analysis was performed in the original space containing all explanatory variables. It confirmed that there was potential for classifying the data into groups in the data from the 17 sensors. This was particularly evident when distinguishing the samples from the primary settling tank from those from other treatment stages.

The random forest model showed that, using the readings from the electronic nose, it is possible to build a model that correctly classifies the vast majority of observations from the sample. It should be noted that although the model obtained 100% correctness on the test data, it was not perfect since the classification in the learning data was not 100%. Therefore, it can be considered that the data that did not enter the learning sample were a perfect fit to the existing model.

The classification of points carried out using the k-median method and random forest reflected the levels of contaminants that characterized the samples described by the classical TOC and TSS indices obtained using standard methods described in the Materials and Methods section.

Considering future directions of research and development, the authors believe that it may be possible to optimize the unsupervised models’ response, for example by application of density-based unsupervised machine learning models. These methods might improve the clustering of the original data into homogenous groups. The development of the supervised model could be impossible in terms of accuracy of the model because it is 100% accurate. However, it is possible to advance the model with regard to the operating speed of the algorithm and the efficiency in using computing resources. Such models are boosting algorithms, which could be used to prepare the software, e.g., in the form of API for the electronic nose.

## Figures and Tables

**Table 1:** Number of observations, cardinality, maximal and average dissimilarity of observations from each cluster, diameter and separation of clusters which were the result of k-medians method.

Cluster Number	Size	Maximal Dissimilarity	Average Dissimilarity	Diameter	Separation
1	12	4.68	2.98	5.87	4.02
2	25	5.47	3.33	7.26	3.71
3	61	4.98	3.55	7.33	1.40
4	54	5.21	3.11	7.39	0.66
5	33	4.85	3.02	6.81	0.66

**Table 2:** Number of correct classifications of observations into clusters formed by the k-median method, relative to reference values in the original dataset.

	Reference	Primary Settling Tank	Mixing Chamber	Inflow into Bioreactor	Outflow from Bioreactor	Secondary Settling Tank
Prediction						
1		12	0	0	0	0
2		25	0	0	0	0
3		0	19	20	10	12
4		0	18	17	16	3
5		0	0	0	11	22

**Table 3:** Performance indicators of multi-class classification on a test set.

Metric	Value
Accuracy	1
Kappa	1
Precision	1
Recall	1
F1 score	1
AUC	1

## Data Availability

All important data are available in the paper.

## References

[1] Dominguez D., Gujer W. (2006). Evolution of a Wastewater Treatment Plant Challenges Traditional Design Concepts. Water Res..

[2] Barbusiński K., Szeląg B., Studziński J. (2020). Simulation of the Influence of Wastewater Quality Indicators and Operating Parameters of a Bioreactor on the Variability of Nitrogen in Outflow and Bulking of Sludge: Data Mining Approach. Desalin. Water Treat..

[3] Tiri A., Belkhiri L., Mouni L. (2018). Evaluation of Surface Water Quality for Drinking Purposes Using Fuzzy Inference System. Groundw. Sustain. Dev..

[4] Carugo D., Octon T., Messaoudi W., Fisher A.L., Carboni M., Harris N.R., Hill M., Glynne-Jones P. (2014). A Thin-Reflector Microfluidic Resonator for Continuous-Flow Concentration of Microorganisms: A New Approach to Water Quality Analysis Using Acoustofluidics. Lab Chip.

[5] Freitag S., Schwaighofer A., Radel S., Lendl B. Ultrasound Manipulation of Bacteria in Drinking Water for Attenuated Total Reflection Infrared (ATR-IR) Spectroscopy. https://publik.tuwien.ac.at/files/publik_277081.pdf.

[6] Bodington V., Langford A., Dooley M., Diamond K. (2009). Cardiff WWTW Aeration Optimisation through Scientific Control.

[7] Drewnowski J., Remiszewska-Skwarek A., Fernandez-Morales F.J. (2018). Model Based Evaluation of Plant Improvement at a Large Wastewater Treatment Plant (WWTP). J. Environ. Sci. Health Part A.

[8] Solon K., Flores-Alsina X., Kazadi Mbamba C., Ikumi D., Volcke E.I.P., Vaneeckhaute C., Ekama G., Vanrolleghem P.A., Batstone D.J., Gernaey K.V. (2017). Plant-Wide Modelling of Phosphorus Transformations in Wastewater Treatment Systems: Impacts of Control and Operational Strategies. Water Res..

[9] Zaborowska E., Czerwionka K., Makinia J. (2017). Strategies for Achieving Energy Neutrality in Biological Nutrient Removal Systems—A Case Study of the Slupsk WWTP (Northern Poland). Water Sci. Technol..

[10] Rosso D., Larson L.E., Stenstrom M.K. (2008). Aeration of Large-Scale Municipal Wastewater Treatment Plants: State of the Art. Water Sci. Technol..

[11] Drewnowski J., Remiszewska-Skwarek A., Duda S., Łagód G. (2019). Aeration Process in Bioreactors as the Main Energy Consumer in a Wastewater Treatment Plant. Review of Solutions and Methods of Process Optimization. Processes.

[12] Thomas O., Théraulaz F., Cerdà V., Constant D., Quevauviller P. (1997). Wastewater Quality Monitoring. TrAC Trends Anal. Chem..

[13] Łagód G., Duda S.M., Majerek D., Szutt A., Dołhańczuk-Śródka A. (2019). Application of Electronic Nose for Evaluation of Wastewater Treatment Process Effects at Full-Scale WWTP. Processes.

[14] Bourgeois W., Burgess J.E., Stuetz R.M. (2001). On-Line Monitoring of Wastewater Quality: A Review. J. Chem. Technol. Biotechnol..

[15] Drewnowski J., Remiszewska-Skwarek A., Fudala-Książek S., Łuczkiewicz A., Kumari S., Bux F. (2019). The Evaluation of COD Fractionation and Modeling as a Key Factor for Appropriate Optimization and Monitoring of Modern Cost-Effective Activated Sludge Systems. J. Environ. Sci. Health Part A.

[16] Persaud K., Dodd G. (1982). Analysis of Discrimination Mechanisms in the Mammalian Olfactory System Using a Model Nose. Nature.

[17] Craven M.A., Gardner J.W., Bartlett P.N. (1996). Electronic Noses—Development and Future Prospects. TrAC Trends Anal. Chem..

[18] Bartlett J.W.G., Bartlett P.N. (2000). Electronic Noses. Principles and Applications. Meas. Sci. Technol..

[19] Wilson A.D., Baietto M. (2009). Applications and Advances in Electronic-Nose Technologies. Sensors.

[20] Karakaya D., Ulucan O., Turkan M. (2020). Electronic Nose and Its Applications: A Survey. Int. J. Autom. Comput..

[21] Dewettinck T., Van Hege K., Verstraete W. (2001). The Electronic Nose as a Rapid Sensor for Volatile Compounds in Treated Domestic Wastewater. Water Res..

[22] Bieganowski A., Jaromin-Gleń K., Guz Ł., Łagód G., Józefaciuk G., Franus W., Suchorab Z., Sobczuk H. (2016). Evaluating Soil Moisture Status Using an E-Nose. Sensors.

[23] Doulamis N., Voulodimos A., Doulamis A., Bimpas M., Angeli A., Bakalos N., Giusti A., Philimis P., Varriale A., Ausili A. (2018). WaterSpy: A High Sensitivity, Portable Photonic Device for Pervasive Water Quality Analysis. Sensors.

[24] Mizaikoff B. (2003). Peer Reviewed: Mid-IR Fiber-Optic Sensors. Anal. Chem..

[25] Ayhan B., Kwan C., Zhou J., Kish L.B., Benkstein K.D., Rogers P.H., Semancik S. (2013). Fluctuation Enhanced Sensing (FES) with a Nanostructured, Semiconducting Metal Oxide Film for Gas Detection and Classification. Sens. Actuators B Chem..

[26] Schmera G., Kwan C., Ajayan P., Vajtai R., Kish L.B. (2008). Fluctuation-Enhanced Sensing: Status and Perspectives. IEEE Sens. J..

[27] Krivetskiy V., Malkov I., Garshev A., Mordvinova N., Lebedev O.I., Dolenko S., Efitorov A., Grigoriev T., Rumyantseva M., Gaskov A. (2017). Chemically Modified Nanocrystalline SnO2-Based Materials for Nitrogen-Containing Gases Detection Using Gas Sensor Array. J. Alloys Compd..

[28] Teterycz H. (2005). Grubowarstwowe Chemiczne Czujniki Gazów Na Bazie Dwutlenku Cyny.

[29] Stuetz R.M., Fenner R.A., Engin G. (1999). Assessment of Odours from Sewage Treatment Works by an Electronic Nose, H_2_S Analysis and Olfactometry. Water Res..

[30] Nake A., Dubreuil B., Raynaud C., Talou T. (2005). Outdoor in Situ Monitoring of Volatile Emissions from Wastewater Treatment Plants with Two Portable Technologies of Electronic Noses. Sens. Actuators B Chem..

[31] Capelli L., Sironi S., Céntola P., Del Rosso R., Il Grande M. (2008). Electronic Noses for the Continuous Monitoring of Odours from a Wastewater Treatment Plant at Specific Receptors: Focus on Training Methods. Sens. Actuators B Chem..

[32] Guz Ł., Łagód G., Jaromin-Gleń K., Guz E., Sobczuk H. (2016). Assessment of Batch Bioreactor Odour Nuisance Using an E-Nose. Desalin. Water Treat..

[33] Guz Ł., Łagód G., Jaromin-Gleń K., Suchorab Z., Sobczuk H., Bieganowski A. (2015). Application of Gas Sensor Arrays in Assessment of Wastewater Purification Effects. Sensors.

[34] Stuetz R.M., Fenner R.A., Engin G. (1999). Characterisation of Wastewater Using an Electronic Nose. Water Res..

[35] Bourgeois W., Stuetz R.M. (2002). Use of a Chemical Sensor Array for Detecting Pollutants in Domestic Wastewater. Water Res..

[36] Bourgeois W., Gardey G., Servieres M., Stuetz R.M. (2003). A Chemical Sensor Array Based System for Protecting Wastewater Treatment Plants. Sens. Actuators B Chem..

[37] Bourgeois W., Hogben P., Pike A., Stuetz R.M. (2003). Development of a Sensor Array Based Measurement System for Continuous Monitoring of Water and Wastewater. Sens. Actuators B Chem..

[38] Onkal-Engin G., Demir I., Engin S.N. (2005). Determination of the Relationship between Sewage Odour and BOD by Neural Networks. Environ. Model. Softw..

[39] Rajagopal R., Ranganathan V. (2017). Evaluation of Effect of Unsupervised Dimensionality Reduction Techniques on Automated Arrhythmia Classification. Biomed. Signal Process. Control.

[40] Martis R.J., Acharya U.R., Min L.C. (2013). ECG Beat Classification Using PCA, LDA, ICA and Discrete Wavelet Transform. Biomed. Signal Process. Control.

[41] Everitt B.S., Landau S., Leese M., Stahl D. (2011). Cluster Analysis.

[42] Wold S., Esbensen K., Geladi P. (1987). Principal Component Analysis. Chemom. Intell. Lab. Syst..

[43] MacQueen J. (1967). Some Methods for Classification and Analysis of Multivariate Observations. Proceedings of the Fifth Berkeley Symposium on Mathematical Statistics and Probability, Volume 1: Statistics.

[44] Eisen M.B., Spellman P.T., Brown P.O., Botstein D. (1998). Cluster Analysis and Display of Genome-Wide Expression Patterns. Proc. Natl. Acad. Sci. USA.

[45] Mette A., Hass J. (2008). Guide to Advanced Software Testing.

[46] Nomura K., Mitchard E.T.A. (2018). More than Meets the Eye: Using Sentinel-2 to Map Small Plantations in Complex Forest Landscapes. Remote Sens..

[47] Henry P. (2008). The Testing Network: An Integral Approach to Test Activities in Large Software Projects.

[48] Borowik P., Adamowicz L., Tarakowski R., Wacławik P., Oszako T., Ślusarski S., Tkaczyk M. (2021). Development of a Low-Cost Electronic Nose for Detection of Pathogenic Fungi and Applying It to Fusarium Oxysporum and Rhizoctonia Solani. Sensors.

[49] Wintjens A.G.W.E., Hintzen K.F.H., Engelen S.M.E., Lubbers T., Savelkoul P.H.M., Wesseling G., van der Palen J.A.M., Bouvy N.D. (2021). Applying the Electronic Nose for Pre-Operative SARS-CoV-2 Screening. Surg. Endosc..

[50] Guney S., Atasoy A., Burget R. (2013). Electronic Nose Odor Classification with Advanced Decision Tree Structures. Radioengineering.

[51] Karami H., Rasekh M., Mirzaee-Ghaleh E. (2020). Application of the E-nose Machine System to Detect Adulterations in Mixed Edible Oils Using Chemometrics Methods. J. Food Process. Preserv..

[52] Kumar K., Pande B.P. (2022). Air Pollution Prediction with Machine Learning: A Case Study of Indian Cities. Int. J. Environ. Sci. Technol..

[53] Braz D.C., Neto M.P., Shimizu F.M., Sá A.C., Lima R.S., Gobbi A.L., Melendez M.E., Arantes L.M.R.B., Carvalho A.L., Paulovich F.V. (2022). Using Machine Learning and an Electronic Tongue for Discriminating Saliva Samples from Oral Cavity Cancer Patients and Healthy Individuals. Talanta.

[54] Hongyang T., Daming H., Xingyi H., Aheto J.H., Yi R., Yu W., Ji L., Shuai N., Mengqi X. (2021). Detection of Browning of Fresh-Cut Potato Chips Based on Machine Vision and Electronic Nose. J. Food Process Eng..

[55] Gradišek A., van Midden M., Koterle M., Prezelj V., Strle D., Štefane B., Brodnik H., Trifkovič M., Kvasić I., Zupanič E. (2019). Improving the Chemical Selectivity of an Electronic Nose to TNT, DNT and RDX Using Machine Learning. Sensors.

[56] Men H., Fu S., Yang J., Cheng M., Shi Y., Liu J. (2018). Comparison of SVM, RF and ELM on an Electronic Nose for the Intelligent Evaluation of Paraffin Samples. Sensors.

[57] Pearson K. (1901). LIII. On Lines and Planes of Closest Fit to Systems of Points in Space. Lond. Edinb. Dublin Philos. Mag. J. Sci..

[58] van der Maaten L., Hinton G. (2008). Visualizing Data Using T-SNE. J. Mach. Learn. Res..

[59] Cominola A., Spang E.S., Giuliani M., Castelletti A., Lund J.R., Loge F.J. (2018). Segmentation Analysis of Residential Water-Electricity Demand for Customized Demand-Side Management Programs. J. Clean. Prod..

[60] Moufid M., Tiebe C., El Bari N., Hamada Fakra D.A., Bartholmai M., Bouchikhi B. (2022). Pollution Parameters Evaluation of Wastewater Collected at Different Treatment Stages from Wastewater Treatment Plant Based on E-Nose and E-Tongue Systems Combined with Chemometric Techniques. Chemom. Intell. Lab. Syst..

[61] Kim S., Brady J., Al-Badani F., Yu S., Hart J., Jung S., Tran T.T., Myung N.V. (2021). Nanoengineering Approaches toward Artificial Nose. Front. Chem..

[62] Hinton G., Roweis S. (2002). Stochastic Neighbor Embedding. Adv. Neural Inf. Process. Syst..

[63] Kullback S., Leibler R.A. (1951). On Information and Sufficiency. Ann. Math. Stat..

[64] Linderman G.C., Rachh M., Hoskins J.G., Steinerberger S., Kluger Y. (2019). Fast Interpolation-Based t-SNE for Improved Visualization of Single-Cell RNA-Seq Data. Nat. Methods.

[65] Li W., Cerise J.E., Yang Y., Han H. (2017). Application of T-SNE to Human Genetic Data. J. Bioinform. Comput. Biol..

[66] Beaulaurier J., Zhu S., Deikus G., Mogno I., Zhang X.S., Davis-Richardson A., Canepa R., Triplett E.W., Faith J.J., Sebra R. (2018). Metagenomic Binning and Association of Plasmids with Bacterial Host Genomes Using DNA Methylation. Nat. Biotechnol..

[67] Driver H.E., Kroeber A.L. (1932). Quantitative Expression of Cultural Relationships.

[68] Arora P., Virmani D., Varshney S. (2016). Analysis of K-Means and K-Medoids Algorithm for Big Data. Procedia Comput. Sci..

[69] Vega M., Pardo R., Barrado E., Debán L. (1998). Assessment of Seasonal and Polluting Effects on the Quality of River Water by Exploratory Data Analysis. Water Res..

[70] Simeonov V., Stratis J.A., Samara C., Zachariadis G., Voutsa D., Anthemidis A., Sofoniou M., Kouimtzis T. (2003). Assessment of the Surface Water Quality in Northern Greece. Water Res..

[71] Beddows D.C.S., Dall’Osto M., Harrison R.M. (2009). Cluster Analysis of Rural, Urban, and Curbside Atmospheric Particle Size Data. Environ. Sci. Technol..

[72] Bergman L.E., Wilson J.M., Small M.J., VanBriesen J.M. (2016). Application of Classification Trees for Predicting Disinfection By-Product Formation Targets from Source Water Characteristics. Environ. Eng. Sci..

[73] Chan J.C.-W., Paelinckx D. (2008). Evaluation of Random Forest and Adaboost Tree-Based Ensemble Classification and Spectral Band Selection for Ecotope Mapping Using Airborne Hyperspectral Imagery. Remote Sens. Environ..

[74] Deepnarain N., Nasr M., Kumari S., Stenström T.A., Reddy P., Pillay K., Bux F. (2019). Decision Tree for Identification and Prediction of Filamentous Bulking at Full-Scale Activated Sludge Wastewater Treatment Plant. Process Saf. Environ. Prot..

[75] Szeląg B., Drewnowski J., Łagód G., Majerek D., Dacewicz E., Fatone F. (2020). Soft Sensor Application in Identification of the Activated Sludge Bulking Considering the Technological and Economical Aspects of Smart Systems Functioning. Sensors.

[76] Lou I., Zhao Y. (2012). Sludge Bulking Prediction Using Principle Component Regression and Artificial Neural Network. Math. Probl. Eng..

[77] Güçlü D., Dursun Ş. (2010). Artificial Neural Network Modelling of a Large-Scale Wastewater Treatment Plant Operation. Bioprocess Biosyst. Eng..

[78] Bagheri M., Mirbagheri S.A., Bagheri Z., Kamarkhani A.M. (2015). Modeling and Optimization of Activated Sludge Bulking for a Real Wastewater Treatment Plant Using Hybrid Artificial Neural Networks-Genetic Algorithm Approach. Process Saf. Environ. Prot..

[79] Breiman L. (2001). Random Forests. Mach. Learn..

[80] Baral P., Haq M.A. (2020). Spatial Prediction of Permafrost Occurrence in Sikkim Himalayas Using Logistic Regression, Random Forests, Support Vector Machines and Neural Networks. Geomorphology.

[81] Breiman L. (1996). Bagging Predictors. Mach. Learn..

[82] Dietterich T.G. (2000). An Experimental Comparison of Three Methods for Constructing Ensembles of Decision Trees: Bagging, Boosting, and Randomization. Mach. Learn..

[83] Breiman L. (1999). Using Adaptive Bagging to Debias Regressions.

[84] Ho T.K. Random Decision Forests. Proceedings of the 3rd International Conference on Document Analysis and Recognition.

[85] Grandini M., Bagli E., Visani G. (2020). Metrics for Multi-Class Classification: An Overview. arxiv.

[86] Hand D.J., Till R.J. (2001). A Simple Generalisation of the Area Under the ROC Curve for Multiple Class Classification Problems. Mach. Learn..

[87] Łagód G., Babko R., Jaromin-Gleń K., Kuzmina T., Bieganowski A. (2016). Biofilm Communities in Successive Stages of Municipal Wastewater Treatment. Environ. Eng. Sci..

[88] TGS—For the Detection of Air Contaminants. Figaro Series Datasheet. http://www.figarosensor.com.

[89] R Core Team (2021). R: A Language and Environment for Statistical Computing.

[90] RStudio Team (2022). RStudio: Integrated Development Environment for R.

[91] Kuhn M. (2022). Caret: Classification and Regression Training.

[92] Kuhn M. (2019). The Caret Package. https://topepo.github.io/caret/.

[93] Maechler M., Rousseeuw P., Struyf A., Hubert M., Hornik K. (2022). Cluster: Cluster Analysis Basics and Extensions. https://cran.r-project.org/web/packages/cluster/index.html.

[94] Krijthe J.H. (2015). Rtsne: T-Distributed Stochastic Neighbor Embedding Using a Barnes-Hut Implementation. https://github.com/jkrijthe/Rtsne.

[95] Wickham H., Averick M., Bryan J., Chang W., McGowan L.D., François R., Grolemund G., Hayes A., Henry L., Hester J. (2019). Welcome to the Tidyverse. J. Open Source Softw..

[96] Wickham H. (2016). Ggplot2: Elegant Graphics for Data Analysis.

[97] Hastie T., Tibshirani R., Friedman J. (2009). The Elements of Statistical Learning.

